# Nursing home-sensitive conditions: analysis of routine health insurance data and modified Delphi analysis of potentially avoidable hospitalizations

**DOI:** 10.12688/f1000research.73875.1

**Published:** 2021-12-01

**Authors:** Sabine Bohnet-Joschko, Maria Paula Valk-Draad, Timo Schulte, Oliver Groene

**Affiliations:** 1 Chair of Management and Innovation in Health Care, Witten/Herdecke University, Witten, 58448, Germany; 2 OptiMedis AG, Hamburg, 20095, Germany

**Keywords:** geriatrics, health services research, potentially avoidable hospitalization, hospitalization, long-term care, nursing homes, nursing home-sensitive conditions

## Abstract

**Background: **Hospitalizations of nursing home residents are associated with various health risks. Previous research indicates that, to some extent, hospitalizations of this vulnerable population may be inappropriate and even avoidable. This study aimed to develop a consensus list of hospital discharge diagnoses considered to be nursing home-sensitive, i.e., avoidable.

**Methods: **The study combined analyses of routine data from six statutory health insurance companies in Germany and a two-stage Delphi panel, enhanced by expert workshop discussions, to identify and corroborate relevant diagnoses. Experts from four different disciplines estimated the proportion of hospitalizations that could potentially have been prevented under optimal conditions.
** **

**Results: **We analyzed frequencies and costs of data for hospital admissions from 242,236 nursing home residents provided by statutory health insurance companies. We identified 117 hospital discharge diagnoses, which had a frequency of at least 0.1%. We recruited experts (primary care physicians, hospital specialists, nursing home professionals and researchers) to estimate the proportion of potentially avoidable hospitalizations for the 117 diagnoses deemed avoidable in two Delphi rounds (n=107 in Delphi Round 1 and n=96 in Delphi Round 2, effective response rate=91%). A total of 35 diagnoses with high and consistent estimates of the proportion of potentially avoidable hospitalizations were identified as nursing home-sensitive. In an expert workshop (n=16), a further 25 diagnoses were discussed that had not reached the criteria, of which another 23 were consented to be nursing home-sensitive conditions. Extrapolating the frequency and mean costs of these 58 diagnoses to the national German context yielded total potentially avoidable care costs of €768,304,547, associated with 219,955 nursing home-sensitive hospital admissions.

**Conclusion: **A total of 58 nursing home-relevant diagnoses (ICD-10-GM three-digit level) were classified as nursing home-sensitive using an adapted Delphi procedure. Interventions should be developed to avoid hospital admission from nursing homes for these diagnoses.

## Introduction

Hospitalizations pose various risks to nursing home residents. Residents may experience reduced functioning upon their return to the nursing home (post-hospital syndrome). ^1^ Hospital-acquired conditions may occur, for example, when specific hospital pathogens lead to infections. ^2^ Adverse drug effects can also develop, such as medication overdose or administration of the wrong medication. ^3^ Cognitively impaired patients often experience loss of orientation as well as confusion in an unfamiliar hospital setting. ^4^ Therefore, there should be intense efforts to avoid unnecessary hospitalizations amongst nursing home residents in health systems across the globe.

The issue of preventing hospital admissions has been intensely discussed regarding ambulatory care-sensitive conditions, for which specific indicator sets have been developed to measure the extent and preventability. ^5^^–^^13^ Ambulatory care (or primary care)-sensitive conditions are those which by expert consensus should not require a hospital admission in the presence of effective primary care. Conditions frequently referred to in existing indicator sets include asthma, chronic obstructive pulmonary disease, congestive heart failure, diabetes mellitus and hypertension, among others. ^14^ Indicator sets to measure ambulatory care-sensitive conditions received substantial attention in policy, research, and clinical practice. ^15^ Such indicator sets may identify variations in regional hospital admission rates, which can lead to investigations about appropriate care structures, as well as interventions to reduce unnecessary hospital admissions.

Whether ambulatory care-sensitive indicator sets can be applied to hospital admissions from nursing homes has been subject to debate. One argument against the use of ambulatory care-sensitive indicator sets is that nursing home populations differ significantly from the general population. The age structure of nursing home residents, the number of comorbidities, the geriatric disease spectrum, as well as the healing process of the elderly population and typical medical interventions, differ from those of community-dwelling residents. ^10^^,^^12^ Moreover, the care setting in long-term care facilities, where care is provided by trained nurses and allied professions 24 hours a day, contrasts with community or outpatient care. The characteristics of the nursing home resident population as well as the long-term care setting influence the type of diagnosis that may require hospital admission, its frequency, and its preventability. Therefore, as existing indicators regarding ambulatory care-sensitive conditions are unlikely to be applicable to long-term care settings or patient populations who are nursing home residents, others have urged the need to develop additional nursing home-sensitive indicator sets. ^16^

Various studies have investigated potentially preventable hospital admissions from the long-term care situations/nursing home setting. ^8^^,^^16^^–^^19^ Earlier studies conducted medical chart reviews and convened panels to gauge the preventability of a hospital admission. Using such an approach, Ouslander *et al.* ^8^ estimated that 67% of hospital admissions in the USA were preventable. Others, such as Walker *et al.* ^17^ in Canada, adopted existing indicator sets for ambulatory care-sensitive hospital admissions, and used administrative databases to calculate that 55% of hospital admissions were preventable. In Germany, Leutgeb *et al.* ^10^ compared ambulatory care-sensitive hospital admission rates amongst nursing home and community-dwelling residents, and found that admission rates were significantly higher amongst nursing home residents. Allers *et al.* cautioned in their systematic review that interventions to reduce hospitalization of nursing home residents should be tailored to health care systems: for policy and clinical practice, it is critical that indicator sets are based on consensus of experts working in the field, that they reflect the characteristic of the national/regional nursing home population and settings, and that they take into account the available health system resources, such as the nursing skills available in the facility, or access to family doctor and specialist visits to the nursing home. ^20^

In order to inform policy debate and practical improvement actions to reduce hospital admissions from nursing homes in Germany, this research project aimed to address the following questions: 1. How often are nursing home residents treated in hospital and what are the main diagnoses and associated costs of these hospital admissions? 2. Which hospital cases are nursing home-sensitive, i.e., at least partially preventable under optimal conditions? 3. What is the impact of the estimated preventability, in case these optimal care conditions existed, at the national level?

## Methods

### Overall approach

For this study, a quantitative mixed-method approach was used, in three consecutive phases. First, an analysis was conducted based on health insurance claims data to identify frequent diagnoses amongst nursing home residents admitted to a hospital. The recommendations of the Working Group for the Survey and Utilization of Secondary Data for the analysis of German health insurance claims data were considered. ^21^ These include data quality issues and recommendations on contractual details between researchers and data owners, among others. Second, a RAND/UCLA Appropriateness Method ^22^ was executed, in which a Delphi expert panel and expert workshop were combined, to reach expert consensus regarding the extent to which hospital admissions might be prevented. A randomized clinical trial was not appropriate for our nursing home resident population: the decision to hospitalize or not, often influences the patient’s survival itself in this vulnerable population. Our method yielded the best available scientific evidence with the collective judgment of experts regarding the appropriateness of hospitalization in nursing home residents. And third, an analysis of routine health insurance data was extrapolated to the total German nursing home resident population, based on which the expenses associated with potentially preventable hospitalizations were estimated. The quantitative data analyses were performed with Microsoft Excel 2016 and IBM SPSS Statistics 26.

### Ethical considerations

An ethical approval was not required and therefore waived. We relied on secondary use of anonymous, aggregated routine data and expert health professionals’ assessments. We did not conduct human research, interventional and non-interventional clinical studies nor clinical trials. We did not collect nor use data in the form of direct health care data, nor did we use (residual) human material for scientific purposes.

All experts provided their written informed consent to participate voluntarily. The Delphi procedure was conducted pseudonymously: towards the end of the data collection, experts were requested to provide their name and email address in a separate database, so that they could be reached for subsequent Delphi rounds and payment of the incentive. Neither the name nor e-mail address given could be linked to the data collection, although the names of the participants were known to the research team. Nonetheless, the assessments towards the potential of preventability were completely anonymized and uninfluenced by the research team. The voluntary nature and pseudonymity of participation was pointed out, together with complete information on the EU General Data Protection Regulation.

### Analysis of routine health insurance data

We obtained claims data from six statutory health insurance companies, which together provide a representative data set of about 29.6% of all nursing home residents in the German population. A data request was agreed between the researchers and health insurance companies, whereupon aggregated data on hospital discharge diagnoses were provided. Included were insured persons living in a nursing home in the calendar year 2017, with a hospital discharge diagnosis (coded as ICD-10-GM, three digits) that occurred in more than 0.1% of this population. Nursing home residents whose insurance period ended in 2017 due to a change in health insurance fund, or who did not have a valid insurance period were excluded from the analysis, to be able to consider insurance utilization without gaps. Insured persons who provided implausible information were also excluded from further analysis. Deceased insured persons, however, were not excluded, because otherwise serious illnesses associated with death in hospital might have been underrepresented, and because nursing home patients have a higher risk of death in hospital than comparable populations. ^23^ For the purposes of this study, nursing home residents are those insured persons for whom a start date prior to Jan 1, 2017, was documented for both a need for long-term care and for full inpatient care in an approved nursing home, pursuant to Section 43 of the German Social Code, Book XI, and who – except for deceased insured persons - consistently had these care services in 2017.

The hospital discharge diagnoses had to have a discharge date within the calendar year 2017. This excluded cases who were admitted to hospital in 2017 or before, but discharged in a later calendar year, which was considered unproblematic as, in contrast, cases were included that were admitted before 2017 but discharged in 2017. In addition, the average costs per hospital case were evaluated. Here, the average total amount paid by social health insurance for the case was used, not just the cost share that would result from the diagnosis-related group ( DRG) of the principal diagnosis. The total amount was evaluated entirely on the discharge date. The list of principal hospital diagnoses was aggregated from all insurance companies and sorted in descending order for the analysis, to determine ICD-10 diagnoses with highest frequency and cost. The exact data request is included in our *Extended data* for this publication (see Data availability section).

### Delphi study and expert workshop

We conducted the modified Delphi study between December 2019 and July 2020 as a two-round online tool followed by an expert workshop in September 2020, combining the strengths of the anonymous questioning of experts with the deeper insight emerging from the discussions at the moderated workshop. The online questionnaire and the expert workshop background information and workshop task are included in the *Extended data.*

In the online tool, experts of four different disciplines (physicians working in outpatient and inpatient care, nursing professionals, and researchers) were asked to estimate the proportion of potentially avoidable hospitalizations identified in the health insurance claims data. The assessment of preventability was under the assumptions of optimal, but still realistic conditions: access to trained personnel, resources and infrastructure for monitoring and nursing, and cooperation agreements with ambulatory general and specialist care providers where needed. For each discharge diagnosis, a short description and a link to the official definition on the German ICD-10 classification were added, to facilitate the assessment for all experts. ICD-10 codes were restricted to the three-digit level to ensure comprehension and usability of the list of diagnoses by all professions. The differentiation for subgroups of the ICD-10 three-digit code was indicated by experts via the numerical estimation of the potential preventability. The experts made their assessments on a scale from 0% to 100% in 5% increments (0% meaning hospitalization was unavoidable, 100% meaning all patients could have been treated in the nursing home). In addition, Delphi participants were provided the opportunity to offer voluntary comments for each ICD-10 code, which became mandatory in the event that experts were unable to give a quantitative estimate of the preventability. LimeSurvey (Version 3.27.20+211012) was selected as the tool for online data collection and was customized specifically for this study. Feedback was obtained and processed from four experts regarding the questionnaire tool, to improve overall presentation and instructions for the experts as well as the presentation of the list of ICD-10 codes. Comprehension and practicability of the online evaluation were tested in a pilot group, technical errors were corrected and ambiguities in the content were clarified.

To recruit the experts, we used the Delphi funnel, a panel management model, according to Donohoe *et al.* ^24^ Funneling constituted in first identifying and selecting potential experts or expert groups, after which we approached them. Additionally, gatekeepers were identified to help pinpoint those individuals who would have knowledge of the topic under study. ^25^ The professional networks of the research team were consulted, and potentially eligible experts were contacted through posts on websites and social media such as LinkedIn, via newsletter announcements, through personal email as well as email distribution lists, on personal recommendation, by phone, and through personal visits.

A Delphi group size depends on group dynamics for arriving a consensus among experts. ^26^ It is also subject to the expected loss-to follow up because of attrition. ^24^ The literature recommends 10 to 18 ^26^ or, in case of high attrition, up to 90 experts ^24^ on a Delphi panel. The Delphi panel should be large enough to reach a sufficient number of perspectives from the “inside”. ^26^ A detailed expert selection criteria list was developed, as purposeful panelist selection can reduce attrition due to loss of interest or frustration. ^24^ For our study, experts should either have practical experience from the sectors involved in the treatment of nursing home residents, play an important role in the decision about hospital treatment, or have published scientific research related to the care of elderly patients. According to these recommendations and in order to ensure a large, methodologically robust and balanced sample of experts for the consensus ratings, we chose to reach out to heterogeneous experts from four different disciplines and planned to recruit 100 experts: 30 outpatient/clinical physicians each, 30 nursing professionals and 10 scientists.

Second, we supported the participants’ engagement by distributing an introductory package along with the invitation to participate in the assessment. ^24^ The preparation of the experts was important in order not to compromise the response rate in future rounds. ^25^ Therefore, before the first Delphi round, experts were informed about the content, the objectives of the research project, what they would be asked to do, how much time they would be expected to contribute, what use would be made of the information they provided, the voluntary nature of participation, and data confidentiality. ^25^ Additional information was also given to pseudonymity of the study (see the Ethical considerations section). After giving their informed consent, experts were invited to participate in this study.

**Figure 1.**
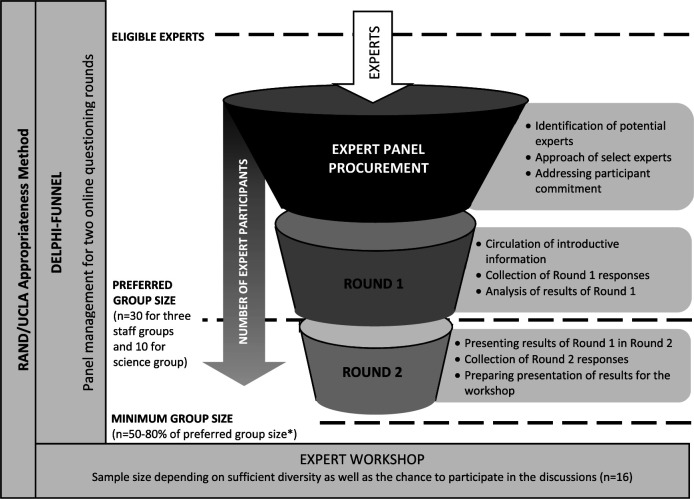
RAND/UCLA-appropriateness method in the nursing home-sensitive conditions study: Delphi expert panel management combined with expert workshop.* *Source: Delphi funnel ^24^; minimum group size. ^32^^–^^35^

Third, results of the first round should be distributed to the panel. ^24^ Therefore, the data collection tool for the second round integrated the results of the expert ratings from the first round. The RAND/UCLA-Appropriateness Method ^22^ is visualized in Figure 1. Expert panel management for the Delphi process was integrated as shown in the Delphi funnel. ^24^

To increase the willingness to participate, a compensation of 100€ was offered for successful participation in both rounds of the survey. For each of the Delphi rounds, the acquisition of experts in that group was discontinued as soon as the required number of participants in each group was reached.

The data from the first Delphi round were analyzed as follows: the median and its interquartile range (containing 50% of all assessments around the median) was reported for each ICD-10-GM three-digit code. Based on this information, the participants were asked to quantify the proportion of potentially avoidable hospitalization again in the second round. If the estimation of the proportion either fell outside the given interquartile range or could not be estimated, the comment field was mandatory again; otherwise, the participants could include extra comments voluntarily. Comments would reveal information about the reasons behind a deviating answer, possible disruptors or problematic conditions complicating the avoidance of hospitalization. Only the comments from the first and second rounds of those ICD-10 codes reaching relevant but dispersed preventability estimates after both online Delphi rounds (conditions described in workshop section) were looked at. This was solely done to be able to identify possible difficulties in the estimation of preventability to be discussed at the expert workshop. Content analysis on this subpart of comments identified themes, which were grouped and described close to the original text, to stimulate the subsequent discussion in the expert workshop. ^27^ We followed SRQR guidelines for this small qualitative component of our research: the analysis of a subpart of free text comments collected as part of our Delphi study. However, this only corresponds to a fraction of our research, the much larger part of our study being based on quantitative consensus techniques and the analysis of administrative health insurance claims data. These were analyzed and reported according to STROBE.

A multidisciplinary expert workshop was convened to discuss those diagnoses, for which the Delphi panel generated highly dispersed data. For face-to-face discussions, it is recommended to have a panel size that permits sufficient diversity, while ensuring that all have a chance to participate. ^22^ This panel consisted of sixteen experts. Again, all four disciplines were represented in equal proportions. A total of 10 out of 16 experts had taken part in the online Delphi rounds. The remaining six experts were provided with the same information on the project from the online rounds. All experts received the same information about the goal and content of the workshop, and all gave their informed consent to take part in course of registration for the online workshop. ICD-10 codes with an assessed preventability potential (median ≥ 75%) and a narrow range of dispersion (dispersion around median ≤ 15%), were considered directly eligible for the list of nursing home-sensitive conditions. In case the preventability potential of at least 75% was *not* comprised in the range of 75% of all expert assessments, the ICD-10 codes were excluded. For all other ICD-10 codes, statistical data (median, modal values, dispersion parameters, bar charts) and categorized comments from the Delphi questionnaire were provided for the expert workshop. The ICD-10 codes were thematically clustered where possible and distributed across three working groups, while the experts were assigned to the working groups according to their expertise.

### Comparing nursing home-sensitive with ambulatory care-sensitive conditions

After preventability estimates were corroborated, the composition and the preventability estimates of these conditions were compared to the results of Sundmacher *et al.* ^7^ They developed a list of ambulatory care-sensitive conditions for the outpatient-German setting. They sorted 258 ICD-10 conditions, expected to be ambulatory care-sensitive by experts, into 40 groups according to disease categories. Each group comprised three- and four-digit ICD-10, because, in some ICD-10, potential preventability was attributed to subcategories only. After that, the potential preventability for each of these groups was estimated. The estimates of preventability for these groups of ICD-10 ranged between 55-96%. ^7^ Of these groups, 22 had an estimated preventability of more than 85% and were posed as core ambulatory care-sensitive conditions. ^7^ The more common ICD-10 discharge diagnoses among nursing home residents, i.e., with a frequency of at least 0.1%, as well as the nursing home-sensitive conditions we found in our study, were then compared to both groups: the ambulatory care-sensitive conditions and the core ambulatory care-sensitive conditions.

### Extrapolating the costs of potentially avoidable hospitalizations

Following the expert consensus process, based on which consensus on a list of nursing home-sensitive hospital admissions was obtained, we used health insurance claims data to calculate the total amount of costs associated with preventable hospital admissions from nursing homes in Germany. We used data from six health insurance companies to assess the total costs associated with the hospitalization case for each diagnosis, and data from the Federal Statistical Office to identify the total number of people living in long-term care facilities in Germany. ^28^ We calculated the number of hospital cases and multiplied those with the average case costs for each diagnosis, which resulted in the total costs of cases with the respective discharge diagnoses. This, further multiplied by the proportion of potential preventability then resulted in the total amount of health care system costs that could potentially be avoided (given optimal care conditions).

## Results

### Analysis of routine health insurance data

We received data from six German statutory health insurance companies according to our data request, covering information on 242,236 nursing home residents. Where data was provided in deviating format, we cleaned and prepared the datasets so they could be aggregated into a global data set. Data included the ICD-10 code, the proportion of hospital admission from nursing-home/long-term care, the gender and age distribution (categorized in 5-year strata) and the mean total cost per case. The age and gender distributions are presented in Table 1 and the gender-differentiated number of hospitalizations, hospitalization proportions and ratios are presented in Table 2. The data received from six health insurance companies were merged and the results are presented in Table 3. This table shows all the hospital discharge diagnoses with a frequency of at least 0.1% in our sample, sorted according to their frequency in descending order, together with a short description of the ICD-10 code, case counts, percentage as well as cumulative percentage of hospital discharge diagnoses from long-term care, and average cost per case for each ICD-10 code.

Over 85% of nursing home residents in our sample were at least 65 years old (206,503 persons of the total sample; Table 1). In the aggregated data set, 44% of fully insured nursing home residents were hospitalized. Thus in 2017, there were 79 hospital admissions for every 100 nursing home residents ( Table 2). The percentage of persons with one or more hospitalizations was slightly higher among men (48%) than among women (44%; Table 2).

**Table 1.** Age and gender distribution of merged health insurance company data of nursing home residents.

Age group (years of age)	Male (N)	Female (N)	Total (N)	Male (%)	Female (%)	Total (%)
0-14	215	323	**538**	0.09%	0.13%	**0.22%**
15-19	459	291	**750**	0.19%	0.12%	**0.31%**
20-24	625	538	**1,163**	0.26%	0.22%	**0.48%**
25-29	1,035	916	**1,951**	0.43%	0.38%	**0.81%**
30-34	1,276	949	**2,225**	0.53%	0.39%	**0.92%**
35-39	1,257	996	**2,253**	0.52%	0.41%	**0.93%**
40-44	1,376	1,019	**2,395**	0.57%	0.42%	**0.99%**
45-49	1,921	1,711	**3,632**	0.79%	0.71%	**1.50%**
50-54	3,112	2,658	**5,770**	1.28%	1.10%	**2.38%**
55-59	3,903	3,292	**7,195**	1.61%	1.36%	**2.97%**
60-64	4,298	3,563	**7,861**	1.77%	1.47%	**3.25%**
65-69	5,011	4,540	**9,551**	2.07%	1.87%	**3.94%**
70-74	5,271	6,638	**11,909**	2.18%	2.74%	**4.92%**
75-79	9,048	16,556	**25,604**	3.74%	6.83%	**10.57%**
80-84	10,454	29,364	**39,818**	4.32%	12.12%	**16.44%**
85-89	9,917	40,872	**50,789**	4.09%	16.87%	**20.97%**
≥90	8,140	60,692	**68,832**	3.36%	25.05%	**28.42%**
**Total**	**67,317**	**174,919**	**242,236**	**27.79%**	**72.21%**	**100.00%**

**Table 2.** Gender-differentiated numbers of hospitalizations, hospitalization proportions and ratios.

	Number of nursing home residents (NHR *) (A)	Proportion of NHR on total sample (A/242,236)	Number of hospitalized NHR (B)	Total number of hospitalizations (C)	Proportion of hospital cases in total sample (B/A)	Ratio of total number of hospitalizations to total number of hospitalized NHR (C/B)
**Male**	67,317	28%	32,275	62,302	48%	1,93
**Female**	174,919	72%	74,679	124,269	43%	1,66
**Total**	242,236	100%	106,954	191,174	44%	1,79

* NHR: nursing home residents.

In total, the top 25 most common discharge diagnoses accounted for 97,378 cases ( Table 3). They covered about half of all hospital cases. About one-third of all hospital cases accounted for one of the following diagnoses: heart failure, pneumonia, fracture of the femur, dehydration, diseases of the urinary tract, intracranial injuries, sepsis, cerebral infarction, and epilepsy. Diseases of the central and peripheral nervous system most frequently led to inpatient treatment of nursing home residents (18%), closely followed by diseases of the respiratory tract (17%) and the digestive tract (15%). The distribution of treatment costs showed a partly different ranking. Neurological diseases such as stroke and epilepsy accounted for the largest share of costs (21%), followed by diseases of the musculoskeletal system (17%), the respiratory system (16%), and the cardiovascular system (13%). From a macroeconomic perspective, it is particularly interesting to note that in the individual organ system groups, a particularly high proportion of costs could be allocated to a few discharge diagnoses. These included fractures in musculoskeletal diseases (over 80%) and pneumonia in respiratory diseases (almost 60%).

**Table 3.** Merged results of analyses on data received from six health insurance companies on nursing home hospitalizations in Germany. *

No.	ICD-10- GM (three digits)	ICD-name	Cases	% hospital cases	% hospital cases (cumulated)	Mean cost per hospital case
1	I50	Heart failure	9,924	5.19%	5.19%	3,683 €
2	J18	Pneumonia (pathogen not specified)	8,503	4.45%	9.64%	3,671 €
3	S72	Fracture of the femur	8,029	4.20%	13.84%	7,794 €
4	E86	Lack of volume	6,579	3.44%	17.28%	2,549 €
5	N39	Other diseases of the urinary system	6,150	3.22%	20.50%	2,613 €
6	S06	Intracranial injury	5,054	2.64%	23.14%	2,104 €
7	A41	Other sepsis	4,508	2.36%	25.50%	4,984 €
8	J69	Pneumonia due to solid and liquid substance	4,486	2.35%	27.85%	4,007 €
9	I63	Cerebral infarction	4,410	2.31%	30.15%	6,228 €
10	G40	Epilepsy	4,149	2.17%	32.32%	3,371 €
11	J44	Other chronic obstructive pulmonary disease	4,125	2.16%	34.48%	4,346 €
12	E11	Diabetes mellitus, type 2	3,173	1.66%	36.14%	4,197 €
13	K56	Paralytic ileus and intestinal obstruction without hernia	2,913	1.52%	37.66%	4,418 €
14	J20	Acute bronchitis	2,563	1.34%	39.00%	2,692 €
15	I70	Atherosclerosis	2,487	1.30%	40.31%	6,912 €
16	K92	Other diseases of the digestive system	2,396	1.25%	41.56%	2,277 €
17	S00	Superficial injury of the head	2,362	1.24%	42.79%	1,122 €
18	N17	Acute renal failure	2,328	1.22%	44.01%	4,375 €
19	S32	Fracture of the lumbar spine and pelvis	2,162	1.13%	45.14%	4,166 €
20	F05	Delirium not caused by alcohol or other psychotropic substance	2,039	1.07%	46.21%	5,915 €
21	J15	Pneumonia due to bacteria, not classified elsewhere	2,012	1.05%	47.26%	4,723 €
22	N30	Cystitis	1,833	0.96%	48.22%	2,771 €
23	A09	Other and unspecified gastroenteritis and colitis of infections and unspecified origin	1,782	0.93%	49.15%	2,210 €
24	R55	Syncope and collapse	1,720	0.90%	50.05%	1,933 €
25	F20	Schizophrenia	1,691	0.88%	50.94%	8,144 €
26	I10	Essential (primary) hypertension	1,673	0.88%	51.81%	2,069 €
27	I21	Acute myocardial infarction	1,612	0.84%	52.66%	5,000 €
28	K80	Cholelithiasis	1,595	0.83%	53.49%	4,847 €
29	K59	Other functional intestinal disorders	1,586	0.83%	54.32%	1,930 €
30	S42	Fracture of shoulder and upper arm	1,407	0.74%	55.06%	4,903 €
31	J22	Unspecified acute lower respiratory infection	1,400	0.73%	55.79%	2,923 €
32	A46	Erysipelas	1,355	0.71%	56.50%	2,894 €
33	G45	Transient cerebral ischemic attacks and related syndromes	1,345	0.70%	57.20%	3,493 €
34	K21	Gastro-esophageal reflux disease	1,328	0.69%	57.89%	2,215 €
35	K29	Gastritis and duodenitis	1,319	0.69%	58.58%	2,314 €
36	N13	Obstructive and reflux uropathy	1,246	0.65%	59.24%	2,813 €
37	I48	Atrial fibrillation and flutter	1,176	0.62%	59.85%	2,959 €
38	D50	Iron deficiency anemia	1,159	0.61%	60.46%	3,035 €
39	S22	Fracture of rib(s), sternum and thoracic spine	1,130	0.59%	61.05%	3,263 €
40	A04	Other bacterial intestinal infections	1,062	0.56%	61.60%	3,950 €
41	L89	Pressure ulcer	1,043	0.55%	62.15%	8,282 €
42	T85	Complications of other internal prosthetic devices, implants and grafts	986	0.52%	62.67%	3,247 €
43	F06	Other mental disorders due to known physiological condition	969	0.51%	63.17%	6,953 €
44	G20	Parkinson's disease	952	0.50%	63.67%	5,167 €
45	C44	Other and unspecified malignant neoplasm of skin	874	0.46%	64.13%	3,017 €
46	T84	Complications of internal orthopedic prosthetic devices, implants and grafts	858	0.45%	64.58%	9,410 €
47	E87	Other disorders of fluid, electrolyte and acidbase balance	857	0.45%	65.02%	2,851 €
48	I80	Thrombosis, phlebitis and thrombophlebitis	847	0.44%	65.47%	2,256 €
49	K57	Diverticular disease of intestine	845	0.44%	65.91%	4,114 €
50	H25	Age-related cataract	830	0.43%	66.34%	1,517 €
51	B99	Other and unspecified infectious diseases	819	0.43%	66.77%	2,632 €
52	G30	Alzheimer’s disease	815	0.43%	67.20%	5,989 €
53	J96	Respiratory failure, not elsewhere classified	761	0.40%	67.60%	9,046 €
54	S82	Fracture of lower leg, including ankle	753	0.39%	67.99%	4,785 €
55	S52	Fracture of forearm	729	0.38%	68.37%	3,218 €
56	I26	Pulmonary embolism	714	0.37%	68.75%	3,917 €
57	N18	Chronic kidney disease (CKD)	714	0.37%	69.12%	4,542 €
58	K22	Other diseases of esophagus	707	0.37%	69.49%	3,759 €
59	G41	Status epilepticus	677	0.35%	69.84%	5,775 €
60	F25	Schizoaffective disorders	663	0.35%	70.19%	8,356 €
61	S70	Superficial injury of hip and thigh	653	0.34%	70.53%	1,510 €
62	R31	Hematuria	650	0.34%	70.87%	2,084 €
63	D64	Other anemias	639	0.33%	71.21%	2,810 €
64	R40	Somnolence, stupor and coma	636	0.33%	71.54%	2,220 €
65	F10	Alcohol related disorders	614	0.32%	71.86%	3,334 €
66	S30	Superficial injury of abdomen, lower back, pelvis and external genitals	602	0.31%	72.17%	1,463 €
67	K52	Other and unspecified noninfective gastroenteritis and colitis	600	0.31%	72.49%	2,600 €
68	T82	Complications of cardiac and vascular prosthetic devices, implants and grafts	594	0.31%	72.80%	5,690 €
69	T83	Complications of genitourinary prosthetic devices, implants and grafts	586	0.31%	73.11%	1,898 €
70	M54	Dorsalgia	576	0.30%	73.41%	2,519 €
71	I61	Nontraumatic intracerebral hemorrhage	561	0.29%	73.70%	7,477 €
72	C34	Malignant neoplasm of bronchus and lung	552	0.29%	73.99%	4,163 €
73	K55	Vascular disorders of intestine	546	0.29%	74.27%	5,838 €
74	R07	Pain in throat and chest	542	0.28%	74.56%	1,260 €
75	R13	Aphagia and dysphagia	517	0.27%	74.83%	2,202 €
76	C50	Malignant neoplasm of breast	507	0.27%	75.09%	4,532 €
77	F33	Major depressive disorder, recurrent	505	0.26%	75.36%	8,731 €
78	K25	Gastric ulcer	504	0.26%	75.62%	4,742 €
79	L03	Cellulitis and acute lymphangitis	487	0.25%	75.88%	2,911 €
80	A08	Viral and other specified intestinal infections	479	0.25%	76.13%	2,687 €
81	I74	Arterial embolism and thrombosis	476	0.25%	76.38%	7,158 €
82	R10	Abdominal and pelvic pain	465	0.24%	76.62%	1,478 €
83	I95	Hypotension	457	0.24%	76.86%	1,802 €
84	T81	Complications of procedures, not elsewhere classified	437	0.23%	77.09%	5,231 €
85	G35	Multiple sclerosis	428	0.22%	77.31%	5,181 €
86	I20	Angina pectoris	427	0.22%	77.53%	2,805 €
87	M80	Osteoporosis with current pathological fracture	424	0.22%	77.76%	4,484 €
88	J10	Influenza due to other identified influenza virus	423	0.22%	77.98%	3,670 €
89	R26	Abnormalities of gait and mobility	413	0.22%	78.19%	4,670 €
90	S02	Fracture of skull and facial bones	406	0.21%	78.41%	2,631 €
91	F01	Vascular dementia	401	0.21%	78.61%	5,352 €
92	R11	Nausea and vomiting	395	0.21%	78.82%	1,786 €
93	C18	Malignant neoplasm of colon	383	0.20%	79.02%	8,317 €
94	K62	Other diseases of anus and rectum	371	0.19%	79.22%	3,256 €
95	K26	Duodenal ulcer	366	0.19%	79.41%	4,268 €
96	J40	Bronchitis, not specified as acute or chronic	365	0.19%	79.60%	2,493 €
97	Z45	Encounter for adjustment and management of implanted device	363	0.19%	79.79%	3,835 €
98	K83	Other diseases of biliary tract	338	0.18%	79.96%	3,987 €
99	C67	Malignant neoplasm of bladder	334	0.17%	80.14%	3,986 €
100	F07	Personality and behavioral disorders	317	0.17%	80.31%	6,327 €
101	A49	Bacterial infection of unspecified site	293	0.15%	80.46%	2,985 €
102	S01	Open wound of head	289	0.15%	80.61%	1,007 €
103	R33	Retention of urine	275	0.14%	80.75%	1,299 €
104	K40	Inguinal hernia	267	0.14%	80.89%	3,366 €
105	S80	Superficial injury of knee and lower leg	258	0.13%	81.03%	2,914 €
106	Z49	Encounter for care involving renal dialysis	252	0.13%	81.16%	8,529 €
107	L02	Cutaneous abscess, furuncle and carbuncle	215	0.11%	81.27%	3,176 €
108	A40	Streptococcal sepsis	212	0.11%	81.38%	4,641 €
109	R06	Abnormalities of breathing	206	0.11%	81.49%	1,265 €
110	I44	Atrioventricular and left bundle-branch block	202	0.11%	81.60%	5,528 €
111	K08	Other disorders of teeth and supporting structures	196	0.10%	81.70%	2,362 €
112	T17	Foreign body in respiratory tract	195	0.10%	81.80%	3,312 €
113	N20	Calculus of kidney and ureter	192	0.10%	81.90%	3,554 €
114	I49	Other cardiac arrhythmias	191	0.10%	82.00%	5,138 €
115	S20	Superficial injury of thorax	188	0.10%	82.10%	1,563 €
116	I35	Nonrheumatic aortic valve disorders	187	0.10%	82.20%	7,909 €
117	F32	Major depressive disorder, single episode	181	0.10%	82.29%	5,245 €
Rest		Over 455 other diagnoses with frequency <0,1%	33,852	17,71%	100%	4,226 €
Total hospitalizations		All hospitalization diagnoses in our study population of 242,236 nursing home residents	191,174	100%	100%	4,030 €

* Table 3 is based on the data request (see *Extended data* in the data availability section).

The average cost of a nursing home resident hospital case in 2017 in the sample was €4,030 (191,174 hospitalizations with hospital costs of €770,368,090; Tables 3 and 7). Extending Table 3 to the defined cut-off point of 0.1% share of all hospital cases, 117 different ICD-10 diagnoses were considered for the Delphi study (totaling 157,322 cases).

### Delphi study and expert workshop

We were able to exceed our recruitment goal of n = 100 experts for the Delphi study, as defined in the protocol, to a number of 107. Of the 107 experts participating in the first round of the Delphi study, 104 (97.2%) had indicated their name and e-mail address and were invited to the second round. Of these, 96 (92.3%) followed the invitation ( Table 4) and 95 completed the second round successfully so that responses were usable (effective response rate 91%). There were no significant differences in age, gender, and years of experience between responders and non-responders.

**Table 4.** Expert panel composition for the Delphi study.

Experience	Expertise/specialization	N planned	N round 1	N round 2
Clinical, physician	Primary care physicians	30	31	29
Clinical, physician	Hospital specialists	30	34	30
Clinical, nursing	Nursing home professionals/staff	30	31	26
Research	Medical research, pharmacology/pharmacy, nursing science, health services research	10	11	11
	Total	100	107	96

Details of the experts’ estimate of the preventability of hospitalizations from the nursing home following the Delphi rounds and the expert workshop are reported in Table 5. In the first Delphi round, experts estimated the proportion of potentially avoidable hospitalizations for 117 ICD-10 codes which were identified in the previous step, based on the analysis of routine health insurance data. Experts were asked to provide their estimations assuming optimal structural and care conditions. Where ICD-10 codes were not assessed by six or more experts, we reviewed the comments to identify why experts had difficulties in assessing the potential preventability of individual ICD-10 codes. This concerned 20 of the 117 ICD-10 codes (17%). To avoid further assessment difficulties, notes were included in the explanations for these ICD-10 codes, or their lay-out was changed for the second Delphi round. Furthermore, ICD-10 codes were planned to be excluded when at least 75% of the experts estimated the preventability as zero in the first round. This condition was never met. Secondly, ICD-10 codes were planned to be combined if the diseases were very similar (in terms of symptoms, diagnosis, prognosis, treatment) and the proportions of potential preventability were nearly identical. Two conditions for “nearly identical potential preventability” had to be fulfilled: the medians of the estimated preventability should not differ by more than 5% and the limits of the interquartile range had to be less than 10% apart). Neither conditions were met sufficiently. Therefore, for the second round, all 117 hospital discharge diagnoses were assessed again.

**Table 5.** Estimation of potential hospitalization preventability of nursing home relevant hospitalizations using a modified Delphi-study, including a comparison to ambulatory care-sensitive conditions.

No.	ICD ^	Short description *in italic the 25 ICD discussed in the expert workshop* nursing home-sensitive condition are highlighted in grey	Avoidability in % consensus validated in expert workshop	R2 *-median Avoida-bility in %	R1 **-Median Avoida-bility in %	R2 *-dispersion interval with 75% of assessments	R2 * Percen-tile 12,5	R2 * Percen- tile 87,5	R2 *- standard deviation	R2 * specialty comparison p-value K-W-test ^1^	N ^+^	Belonging to any of the 40 ambulatory care-sensitive condition groups ^++^	Belonging to any of the 22 core ambulatory care-sensitive condition groups ^++^	Preventa-bility in % of core ambulatory care-sensitive condition groups ^+++^
*1.*	*L02*	*Cutaneous abscess, furuncle and carbuncle*	*100*	*70*	*70*	*15.0*	*70.00*	*85.00*	*8.291*	** *0.008*	*95*	*yes*	*yes*	*77%*
*2.*	*H25*	*Age-related cataract*	*95*	*85*	*85*	*50.0*	*40.00*	*90.00*	*20.065*	** *0.009*	*95*	*yes*	*yes*	*81%*
3.	E11	DM Typ Type 2 diabetes mellitus2	90	90	90	5.0	90.00	95.00	4.756	0.489	94	yes	yes	81%
4.	F01	Vascular dementia	90	90	90	5.0	90.00	95.00	4.475	*0.066	95	yes	no	
*5.*	*F07*	*Personality and behavioral disorders*	*90*	*75*	*75*	*20.0*	*65.00*	*85.00*	*10.226*	* *0.091*	*95*	*no*	*no*	
6.	G30	Alzheimer’s disease	90	90	90	10.0	90.00	100.00	6.836	**0.019	95	no	no	
*7.*	*G40*	*Epilepsy and recurrent seizures*	*90*	*70*	*70*	*20.0*	*65.00*	*85.00*	*10.903*	** *0.001*	*95*	*no*	*no*	
8.	I10	Essential (primary) hypertension	90	90	90	5.0	90.00	95.00	3.969	0.147	95	yes	yes	83%
9.	J20	Acute bronchitis	90	90	90	15.0	80.00	95.00	7.937	*0.050	95	yes	yes	76%
10.	J40	Bronchitis, not specified as acute or chronic	90	90	90	15.0	80.00	95.00	5.740	**0.030	94	yes	yes	76%
11.	K21	Gastro-esophageal reflux disease	90	90	90	5.0	90.00	95.00	4.371	0.201	95	yes	no	
12.	K59	Other functional intestinal disorders	90	90	90	5.0	90.00	95.00	3.807	0.238	95	partly	partly	77%
13.	N30	Cystitis	90	90	90	10.0	90.00	100.00	5.115	**0.040	95	yes	yes	86%
14.	S00	Superficial injury of head	90	90	90	5.0	90.00	95.00	4.524	*0.092	95	no	no	
15.	S20	Superficial injury of thorax	90	90	90	5.0	90.00	95.00	5.970	*0.089	95	no	no	
16.	S80	Superficial injury of knee and lower leg	90	90	90	5.0	90.00	95.00	5.880	0.157	95	no	no	
17.	F32	Major depressive disorder, single episode	85	85	85	10.0	85.00	95.00	5.919	0.483	95	yes	yes	70%
18.	F33	Major depressive disorder, recurrent	85	85	85	10.0	85.00	95.00	6.425	0.161	95	yes	yes	70%
19.	I95	Hypotension	85	85	85	15.0	80.00	95.00	7.746	*0.097	95	yes	yes	76%
*20.*	*K08*	*Other disorders of teeth and supporting structures*	*85*	*85*	*85*	*20.0*	*75.00*	*95.00*	*10.906*	** *0.021*	*95*	*yes*	*yes*	*94%*
21.	L89	Pressure ulcer	85	85	85	15.0	80.00	95.00	6.061	**0.000	95	yes	no	
22.	M54	Dorsalgia	85	85	85	10.0	85.00	95.00	4.657	**0.048	95	yes	yes	81%
*23.*	*N18*	*Chronic kidney disease (CKD)*	*85*	*70*	*70*	*15.0*	*65.00*	*80.00*	*8.470*	*0.594*	*95*	*no*	*no*	
24.	N39	Other disorders of urinary system	85	85	85	10.0	85.00	95.00	7.304	**0.048	95	partly	partly	86%
25.	R26	Abnormalities of gait and mobility	85	85	85	11.3	79.38	90.63	8.047	0.118	94	no	no	
26.	S70	Superficial injury of hip and thigh	85	85	85	5.0	85.00	90.00	5.323	0.103	95	no	no	
*27.*	*A04*	*Other bacterial intestinal infections*	*80*	*70*	*70*	*15.0*	*65.00*	*80.00*	*9.244*	*0.514*	*95*	*yes*	*yes*	*75%*
28.	A08	Viral and other specified intestinal infections	80	80	80	15.0	75.00	90.00	7.343	*0.095	95	yes	yes	75%
29.	A09	Infectious gastroenteritis and colitis, unspecified	80	80	80	10.0	75.00	85.00	5.661	0.603	95	yes	yes	75%
30.	A46	Erysipelas	80	80	80	15.0	75.00	90.00	8.445	*0.051	95	yes	yes	77%
31.	D50	Iron deficiency anemia	80	80	80	10.6	80.00	90.63	7.373	**0.002	94	yes	yes	85%
32.	D64	Other anemias	80	80	80	5.0	80.00	85.00	7.399	0.169	95	no	no	
*33.*	*F10*	*Alcohol related disorders*	*80*	*60*	*60*	*25.0*	*50.00*	*75.00*	*12.371*	* *0.069*	*94*	*yes*	*yes*	*66%*
34.	G20	Parkinson's disease	80	80	80	15.0	75.00	90.00	6.999	**0.012	94	no	no	
35.	I70	Atherosclerosis	80	80	80	10.0	80.00	90.00	7.254	**0.022	94	yes	yes	76%
36.	J22	Unspecified acute lower respiratory infection	80	80	80	15.0	70.00	85.00	8.336	0.115	95	no	no	
37.	K29	Gastritis and duodenitis	80	80	80	10.0	80.00	90.00	8.407	0.120	95	partly	no	
38.	R11	Nausea and vomiting	80	80	80	10.0	80.00	90.00	6.505	**0.007	94	no	no	
*39.*	*R13*	*Aphagia and dysphagia*	*80*	*60*	*60*	*25.6*	*54.38*	*80.00*	*12.425*	** *0.005*	*94*	*no*	*no*	
40.	S30	Superficial injury of abdomen, lower back, pelvis and external genitals	80	80	80	10.0	80.00	90.00	7.130	0.166	95	no	no	
*41.*	*C44*	*Other and unspecified malignant neoplasm of skin*	*75*	*60*	*60*	*25.0*	*50.00*	*75.00*	*13.044*	* *0.060*	*95*	*yes*	*no*	
*42.*	*E86*	*Volume depletion*	*75*	*70*	*70*	*20.0*	*65.00*	*85.00*	*13.584*	** *0.030*	*95*	*yes*	*no*	
*43.*	*F05*	*Delirium due to known physiological condition*	*75*	*50*	*50*	*40.0*	*35.00*	*75.00*	*16.689*	*0.112*	*95*	*no*	*no*	
*44.*	*F06*	*Other mental disorders due to known physiological condition*	*75*	*70*	*70*	*20.0*	*60.00*	*80.00*	*10.980*	*0.360*	*95*	*no*	*no*	
*45.*	*F20*	*Schizophrenia*	*75*	*55*	*55*	*25.0*	*50.00*	*75.00*	*11.787*	*0.235*	*95*	*no*	*no*	
46.	G35	Multiple sclerosis	75	75	75	15.0	70.00	85.00	8.045	**0.004	95	no	no	
*47.*	*I50*	*Heart failure*	*75*	*60*	*60*	*15.0*	*60.00*	*75.00*	*9.699*	** *0.007*	*95*	*yes*	*yes*	*64%*
*48.*	*I80*	*Thrombosis, phlebitis and thrombophlebitis*	*75*	*55*	*55*	*25.0*	*50.00*	*75.00*	*13.516*	** *0.001*	*95*	*partly*	*partly*	*76%*
*49.*	*J10*	*Influenza due to other identified influenza virus*	*75*	*65*	*65*	*25.0*	*55.00*	*80.00*	*11.741*	* *0.059*	*95*	*yes*	*yes*	*68%*
50.	J44	Other chronic obstructive pulmonary disease	75	75	75	15.0	70.00	85.00	8.638	**0.006	95	yes	yes	76%
*51.*	*K25*	*Gastric ulcer*	*75*	*65*	*65*	*20.0*	*55.00*	*75.00*	*13.734*	*0.154*	*95*	*partly*	*no*	
*52.*	*K26*	*Duodenal ulcer*	*75*	*65*	*65*	*15.0*	*60.00*	*75.00*	*12.768*	** *0.009*	*95*	*no*	*no*	
53.	K52	Other and unspecified noninfective gastroenteritis and colitis	75	75	75	15.0	70.00	85.00	7.560	**0.011	95	partly	partly	77%
54.	K57	Diverticular disease of intestine	75	75	75	15.0	75.00	90.00	11.201	**0.017	95	yes	yes	77%
*55.*	*K62*	*Other diseases of anus and rectum*	*75*	*70*	*70*	*20.0*	*60.00*	*80.00*	*7.877*	*0.291*	*95*	*no*	*no*	
*56.*	*S01*	*Open wound of head*	*75*	*65*	*65*	*20.0*	*55.00*	*75.00*	*11.928*	*0.142*	*95*	*no*	*no*	
*57.*	*E87*	*Other disorders of fluid, electrolyte and acidbase balance*	*70*	*60*	*60*	*25.0*	*50.00*	*75.00*	*10.543*	*0.226*	*95*	*partly*	*no*	
*58.*	*R07*	*Pain in throat and chest*	*70*	*60*	*60*	*26.3*	*53.75*	*80.00*	*12.151*	* *0.066*	*93*	*no*	*no*	*77%*
*59.*	*F25*	*Schizoaffective disorders*	*65*	*65*	*65*	*25.0*	*50.00*	*75.00*	*10.865*	*0.225*	*94*	*no*	*no*	
60.	B99	Other and unspecified infectious diseases	60	60	60	20.0	50.00	70.00	9.124	**0.001	95	no	no	
*61.*	*L03*	*Cellulitis and acute lymphangitis*	*60*	*60*	*60*	*25.0*	*50.00*	*75.00*	*11.256*	** *0.003*	*95*	*no*	*no*	
62.	R31	Hematuria	55	55	55	25.0	45.00	70.00	11.094	**0.041	95	no	no	
63.	A49	Bacterial infection of unspecified site	50	50	50	15.0	50.00	65.00	9.205	**0.002	95	no	no	
64.	I48	Atrial fibrillation and flutter	50	50	50	26.3	44.38	70.63	14.112	**0.011	94	no	no	
65.	J15	Bacterial pneumonia, not elsewhere classified	50	50	50	30.0	35.00	65.00	11.752	*0.074	95	partly	partly	
66.	J18	Pneumonia, unspecified organism	50	50	50	30.0	40.00	70.00	13.368	*0.072	95	partly	partly	
67.	K80	Cholelithiasis	50	50	50	25.0	40.00	65.00	12.742	0.886	95	no	no	
68.	R06	Abnormalities of breathing	50	50	50	25.0	45.00	70.00	13.344	0.150	93	no	no	
69.	R10	Abdominal and pelvic pain	50	50	50	20.6	45.00	65.63	9.468	0.525	94	no	no	
70.	R33	Retention of urine	50	50	50	30.0	40.00	70.00	14.873	0.397	95	no	no	
71.	R55	Syncope and collapse	50	50	50	25.0	40.00	65.00	13.069	0.173	94	no	no	
72.	Z45	Encounter for adjustment and management of implanted device	50	50	50	30.0	35.00	65.00	15.483	0.668	95	no	no	
73.	I35	Nonrheumatic aortic valve disorders	45	45	45	20.0	35.00	55.00	11.365	0.179	95	no	no	
74.	I44	Atrioventricular and left bundle-branch block	45	45	45	30.0	30.00	60.00	14.344	**0.044	95	no	no	
75.	Z49	Encounter for care involving renal dialysis	45	45	45	45.0	20.00	65.00	20.788	*0.058	95	no	no	
76.	I20	Angina pectoris	40	40	40	20.0	30.00	50.00	10.836	0.871	94	yes	yes	
77.	K22	Other diseases of esophagus	40	40	40	35.0	15.00	50.00	14.402	0.290	95	no	no	
78.	K40	Inguinal hernia	40	40	40	25.0	25.00	50.00	12.341	0.416	95	no	no	
79.	M80	Osteoporosis with current pathological fracture	40	40	40	40.0	20.00	60.00	14.826	0.356	95	no	no	
80.	I49	Other cardiac arrhythmias	35	35	35	20.0	30.00	50.00	11.811	0.709	95	partly	partly	
81.	J96	Respiratory failure, not elsewhere classified	35	35	35	25.0	25.00	50.00	13.100	0.159	95	no	no	
82.	J69	Pneumonitis due to solids and liquids	30	30	30	35.0	15.00	50.00	14.566	0.521	95	no	no	
83.	K55	Vascular disorders of intestine	30	30	30	15.0	15.00	30.00	9.732	0.378	95	no	no	
84.	N13	Obstructive and reflux uropathy	30	30	30	20.0	20.00	40.00	10.274	0.212	95	no	no	
85.	C34	Malignant neoplasm of bronchus and lung	25	25	25	25.0	10.00	35.00	10.775	0.712	94	no	no	
86.	C50	Malignant neoplasm of breast	25	25	25	20.0	10.00	30.00	10.254	**0.038	95	no	no	
87.	C67	Malignant neoplasm of bladder	25	25	25	20.0	10.00	30.00	10.896	0.138	95	no	no	
88.	N20	Calculus of kidney and ureter	25	25	25	10.6	15.00	25.63	7.121	0.569	94	no	no	
89.	C18	Malignant neoplasm of colon	20	20	20	15.0	10.00	25.00	8.405	*0.057	94	no	no	
90.	S22	Fracture of rib(s), sternum and thoracic spine	20	20	20	20.0	10.00	30.00	12.515	0.121	95	no	no	
91.	T81	Complications of procedures, not elsewhere classified	20	20	20	20.0	10.00	30.00	12.269	*0.089	94	no	no	
92.	G45	Transient cerebral ischemic attacks and related syndromes	15	15	15	20.0	0.00	20.00	8.960	*0.072	95	no	no	
93.	K83	Other diseases of biliary tract	15	15	15	15.0	5.00	20.00	6.283	0.427	95	no	no	
94.	K92	Other diseases of digestive system	15	15	15	15.0	5.00	20.00	7.035	*0.079	95	no	no	
95.	S52	Fracture of forearm	15	15	15	40.0	5.00	45.00	14.808	0.491	95	no	no	
96.	I74	Arterial embolism and thrombosis	10	10	10	5.0	5.00	10.00	5.188	0.829	95	no	no	
97.	R40	Somnolence, stupor and coma	10	10	10	20.0	0.00	20.00	12.555	0.473	94	no	no	
98.	S06	Intracranial injury	10	10	10	10.0	5.00	15.00	7.781	0.571	95	no	no	
99.	S32	Fracture of lumbar spine and pelvis	10	10	10	15.0	5.00	20.00	10.091	**0.019	95	no	no	
100.	S42	Fracture of shoulder and upper arm	10	10	10	15.0	5.00	20.00	10.565	0.477	95	no	no	
101.	T17	Foreign body in respiratory tract	10	10	10	10.0	5.00	15.00	10.177	0.796	95	no	no	
102.	T83	Complications of genitourinary prosthetic devices, implants and grafts	10	10	10	10.0	5.00	15.00	7.013	0.334	95	no	no	
103.	T84	Complications of internal orthopedic prosthetic devices, implants and grafts	10	10	10	10.0	5.00	15.00	6.773	0.426	95	no	no	
104.	T85	Complications of other internal prosthetic devices, implants and grafts	10	10	10	10.0	5.00	15.00	6.518	0.279	95	no	no	
105.	G41	Status epilepticus	5	5	5	20.0	0.00	20.00	8.952	0.466	95	no	no	
106.	I26	Pulmonary embolism	5	5	5	10.0	0.00	10.00	12.413	0.185	95	no	no	
107.	I61	Nontraumatic intracerebral hemorrhage	5	5	5	10.0	0.00	10.00	4.982	0.188	95	no	no	
108.	I63	Cerebral infarction	5	5	5	10.0	0.00	10.00	4.244	**0.027	95	no	no	
109.	K56	Paralytic ileus and intestinal obstruction without hernia	5	5	5	5.0	0.00	5.00	3.664	0.220	95	no	no	
110.	N17	Acute kidney failure	5	5	5	15.0	0.00	15.00	11.991	**0.004	95	no	no	
111.	S02	Fracture of skull and facial bones	5	5	5	10.0	0.00	10.00	4.896	*0.091	95	no	no	
112.	S72	Fracture of femur	5	5	5	5.0	0.00	5.00	4.972	0.533	95	no	no	
113.	S82	Fracture of lower leg, including ankle	5	5	5	10.0	0.00	10.00	3.905	0.113	95	no	no	
114.	T82	Complications of cardiac and vascular prosthetic devices, implants and grafts	5	5	5	10.0	0.00	10.00	4.120	*0.063	95	no	no	
115.	A40	Streptococcal sepsis	0	0	0	5.0	0.00	5.00	4.283	0.882	95	no	no	
116.	A41	Other sepsis	0	0	0	5.0	0.00	5.00	4.168	0.868	95	no	no	
117.	I21	Acute myocardial infarction	0	0	0	5.0	0.00	5.00	3.061	0.212	95	no	no	

1 p-value of the Kruskal-Wallis test; H0: There was no difference in the department-specific assessment of avoidance potential.

* 21 ICD-10-GM three-digit tending to have statistically significantly different estimates of potentially avoidable hospitalization by specialty: 0.05≤Kruskall-Wallis p-value<0.10.

** 34 ICD-10-GM triplicates with statistically significantly different assessment of potential avoidable hospitalization by specialties: Kruskall-Wallis p-value<0.05.

^ ICD: International Classification of Diseases, 10th revision, German Version (ICD-10-GM), a direct translation in German language of the ICD-10 of WHO.

° R2: second Delphi round.

°° R1: first Delphi round.

+ N: Number of assessments in the second Delphi round.

++ 258 ambulatory care-sensitive ICD-10 conditions, comprising three- and four-digit codes, grouped according to disease categories in 40 groups; estimated preventability between 55-96%. ^7^

+++ 22 core ambulatory care-sensitive condition groups; estimated preventability at least 85%. ^7^

Comparing the responses to the Delphi rounds, we found no differences in the median estimates of the preventability of the 117 ICD-10 codes; however, the scatter range decreased significantly. The width of the interquartile range decreased, for all ICD-10 codes together, from 42.3% to 5.5% on average. Comparing the respective participants’ assessments in both online questionnaire Delphi rounds, both assessments were very close together: the median difference between round 1 and 2 was maximum 5% for 114 of the 117 ICD-10 codes, and maximum 10% for the three remaining ICD-10 codes.

For 34 of the 117 ICD-10 codes, the four groups of experts gave statistically significant different preventability estimations. On average, estimations differed by only 5%, and by a maximum of 15% for individual ICD-10 codes. In most of these 34 cases, the clinicians indicated slightly lower estimates. A statistically significant difference of 5% in the median between men (median estimate at 25%) and women (median estimate at 30%) was found for only one of the 117 ICD-10 codes. All age groups of experts (under 40 years old (n = 29), 40-49 years old (n = 20), 50-59 years old (n = 25), and 60 years or older (n = 20)) were consistent in their estimates of potential avoidability. For 114 of 117 ICD-10 codes the difference in the estimated proportion of potentially avoidable hospitalizations between individual age groups (median) amounted to maximum 5%. The maximum difference found was 10% (only for one ICD-10 code). Age groups differed in their estimates for six conditions, although for these the average assessments were only 2.5 % and never more than 7.5% apart.

For 38 ICD-10 codes, the potentially avoidable hospitalization rate was estimated to be at least 75%; for 12 ICD-10 codes it was estimated to be at least 90%. For 35 of these 38 ICD-10 codes, the unambiguous assessment of a condition as potentially nursing home-sensitive was already clear after the second round of questioning: the range of dispersion around the median of three quarters of all expert’s assessments for the respective ICD-10 code was 15% or less. The three for which the latter did not apply were discussed at the expert workshop. Further, a total of 22 ICD-10 codes had a median preventability proportion below 75%, but their scatter spectrum for three quarters of all assessments contained the relevant preventability proportion of 75%, signaling a relevant but still ambiguous preventability proportion. Thus, a total of 25 ICD-10 codes were prepared for the expert workshop consensus process ( Table 5; ICD-10 codes discussed in workshop are shown in italic font).

Overall, after the workshop with 16 experts, all proportions for potentially avoidable hospitalizations could be corroborated, and 58 ICD-10 codes with an estimated potential avoidability of at least 70%, were selected for the list of nursing home-sensitive conditions. In Table 5 the nursing home-sensitive conditions are shown in the greyed table part. Table 6 shows these conditions sorted by disease category.

**Table 6.** List of nursing home-sensitive hospital conditions sorted by disease category.

No.	Condition of nursing home-sensitive hospital admission	ICD-10 codes ^	Number of ICDs ^
1	Diabetes Mellitus Type 2	E11	1
2	Volume depletion and other disorders of electrolyte and acid-base balance	E86, E87	2
3	Gastrointestinal ulcers and inflammation, esophageal reflux disease, functional bowel disorders, dysphagia	K08, K21, K25, K26, K29, K52, K57, K59, K62, R13	10
4	Intestinal infections	A04, A08, A09, R11	4
5	Chronic kidney disease, cystitis, other diseases of the urinary system.	N18, N30, N39	3
6	Dementia (vascular or Alzheimer's disease, primary Parkinson's syndrome)	F01, G20, G30	3
7	Mental disorders, personality or behavioral disorders, depressive disorder, schizoaffective disorder and schizophrenia, delirium	F05, F06, F07, F10, F20, F32, F33	7
8	Other diseases of the nervous system (MS, epilepsy)	G35, G40	2
9	Cataracta Senilis	H25	1
10	Anemias of different origin	D50, D64	2
11	Hypertension, hypotension, atherosclerosis, heart failure, thrombosis, phlebitis and thrombophlebitis.	I10, I50, I70, I80, I95	5
12	Other acute/chronic lower respiratory tract infections and influenza, sore throat and chest pain.	J10, J20, J22, J40, J44, R07	6
13	Skin infections, decubital ulcer and pressure zone, skin cancer exclusive melanoma	A46, C44, L02, L89	4
14	Back pain, disturbances of gait and mobility	M54, R26	2
15	Superficial injuries of various parts of the body	S00, S01, S20, S30, S70, S80	6
	Total number of nursing home sensitive conditions		58

^ ICD: International Classification of Diseases, 10th revision, German Version (ICD-10-GM), a direct translation in German language of the ICD-10 of WHO.

### Comparison of nursing home-sensitive with ambulatory care-sensitive conditions

For the comparison of nursing home-sensitive diagnoses with ambulatory care-sensitive diagnoses, the occurrence of the 117 ICD-10 codes in the ambulatory care-sensitive groups was reviewed. In Table 5, the last three columns were added to show the results of this review. Comparing the 58 nursing home-sensitive conditions with the ambulatory care-sensitive conditions, it appeared that only 28 three-digit and seven four-digit nursing home-sensitive ICD-10 conditions were also included in the ambulatory care-sensitive ones. Therefore, 60% (35/58) of the nursing home-sensitive conditions were also partly or completely ambulatory care-sensitive, and 40% were not.

Extending this comparison to all common conditions in nursing home residents, only 29 three-digit and another 10 four-digit of the 117 ICD-10 codes were ambulatory care-sensitive. Thus, only 33% (39/117) of the ICD-10 codes relevant to the nursing home population were partially or wholly ambulatory care-sensitive, and 67% were not.

Sundmacher published preventability estimates for the core ambulatory care-sensitive groups of ICD-10 codes. A total of 27 (47%) nursing home-sensitive conditions belong to these core ambulatory care-sensitive conditions (23 completely [three-digit ICD-10] and four only partially [four-digit ICD-10]), and 53% do not. Of all 117 ICD-10 codes, 31 (26%) conditions appeared partly (seven four-digit ICD-10 codes) or wholly (24 three-digit ICD-10 codes) in this core list, and 74% do not.

On the other hand, 178 of the 258 ambulatory care-sensitive hospitalizations (three- and four-digit level) were not listed in the nursing home-sensitive list (69%).

The preventability estimates were only known to the authors for the core ambulatory care-sensitive condition groups. Therefore, for only 31 out of 117 ICD-10 common nursing home hospitalizations, the preventability potential for the nursing home common as well as nursing home-sensitive ICD-10 codes were compared to their counterpart in the core ambulatory care-sensitive groups. For the 27 nursing home-sensitive conditions, the minimum and maximum differences between both settings was −9% and +23%, respectively. For 12 ICD-10 codes, the preventability potentials of both lists were only 5% apart. For the remaining four nursing home common conditions, the difference was −18% to −41% apart.

Thus, our results show that nursing home-sensitive conditions are to be distinguished from ambulatory care-sensitive conditions: both diagnoses and preventability estimates differed between the settings.

### Extrapolation of costs of potentially avoidable hospitalizations

In 2017, there were 3.4 million persons in need of long-term care in Germany, of which 818,289 were nursing home residents. ^28^ With 242,236 insured persons in inpatient care, our study population represented about 29.6% of all nursing home residents in Germany. Our sample yielded 191,174 hospital cases in one year. The annual incidence was therefore around 0.79 hospital cases per insured person in stationary care (191,174/242,236). Table 7 shows the extrapolation of the results from the analysis of routine health insurance data for Germany. For this purpose, the total costs per ICD-10 code were first calculated, weighted according to their proportion of cases (number of cases*cost per hospital case). The total cost for each ICD-10 code was than multiplied by the extrapolation factor and summed to obtain the total costs incurred in Germany. Calculating the costs for nursing home-sensitive conditions was done accordingly. Total costs per nursing home-sensitive condition were multiplied by the proportion (in %) of potentially avoidable hospitalizations agreed to during the Delphi process, and then summed to allow a weighted calculation by case proportion and prevention potential. This was done to estimate the number of cases and costs that would potentially be avoidable, if certain cross-sectoral and structural conditions were in place for the provision of needs-based care for nursing home residents. Table 7 shows the sample results, the extrapolation for Germany and finally the calculations respective to nursing home-sensitive conditions.

**Table 7.** Extrapolation of the costs based on the analysis of routine health insurance data for Germany. *

Selected ICD-10 code ^ details		Number of hospital cases	Hospitalization costs
For all ICD-10 codes	for our sample	191,174	€770,368,090
	for Germany	645,798	€2,602,353,631
Only for the 117 ICD-10 codes included in the online tool	for our sample *(% of all ICD-10 codes)*	157,322 *(82.29%)*	€632,505,610 *(82.10%)*
	for Germany	531,444	€2,136,645,184
Only for the 58 ICD-10 codes consented as nursing home sensitive conditions	for our sample *(% of all ICD-10 codes) (% of 117 included ICD-10 codes)*	79,979 *(41.84%) (50.84%)*	€281,713,203 *(36.57%) (44.54%)*
	for Germany	270,174	€951,654,564
Of which are potentially avoidable	for our sample	65,133	€227,439,230
	for Germany	219,955	€768,304,547

* Multiplication factor for the extrapolation of results of the health insurance data analysis to the situation in Germany: number of nursing home residents in Germany/sample size (818,289/242,236).

^ ICD: International Classification of Diseases, 10th revision, German Version (ICD-10-GM), a direct translation in German language of the ICD-10 of WHO.

**Figure 2.**
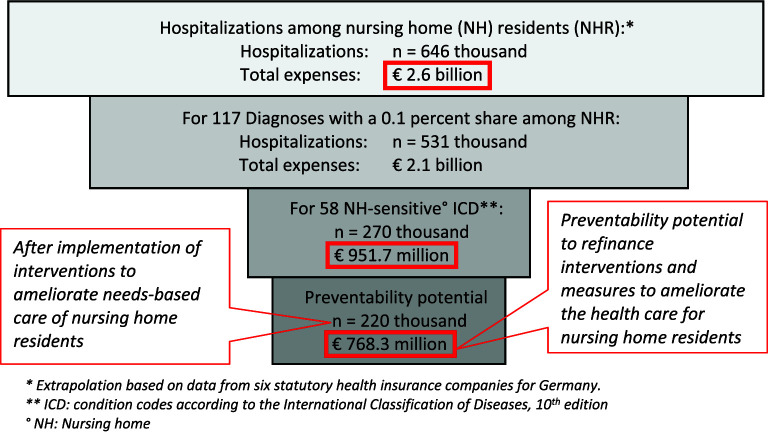
Relevance of nursing home-sensitive conditions for the German health care setting.

The extrapolation forecasts a total of about 646,000 hospital cases per year for all nursing home residents in Germany (818,289/242,236*191,174) with a total cost expense for hospital admissions in the nursing home population of over 2,600,000,000€ (2,6 billion €). Approximately 220,000 hospitalizations might have been prevented if interventions were implemented in favor of greater needs-based care for nursing home residents. If measures were effective, the expenses required to establish them could be met from the funds saved (about three quarters of a billion Euros). The relevance of common nursing home hospitalizations as well as nursing home-sensitive conditions is shown in Figure 2.

## Discussion

### Key findings

We used routine health insurance data from 242,236 nursing home residents to assess frequencies and costs of hospital admissions amongst nursing home residents; we identified 117 hospital discharge diagnoses which had a frequency rate of at least 0.1%. In a two-stage Delphi study, 107 and 96 experts in round 1 and 2, respectively, estimated the potential to avoid a hospitalization for these diagnoses. After two Delphi rounds and an expert workshop, we were able to identify 58 diagnoses considered to be nursing home-sensitive conditions in the context of the German health care system. The frequency of hospital admissions for these diagnoses and associated costs to the care system were substantial, and warrant further discussion on strategies to decrease hospitalizations for these diagnoses.

### Comparison to other studies

There are few comparable studies on nursing home-sensitive hospitalizations. Most of the research in this field focused on ambulatory care-sensitive hospital admissions, which cannot be directly applied to the nursing home context as the nursing home population differs regarding frequency of diseases, healing process, nursing care and systematic care conditions. However, the seminal studies by Purdy *et al.,* ^5^ Ouslander *et al.* ^8^ and Walker *et al.* ^17^ are noteworthy, for developing the concept of avoidable hospital admissions and using various methods established in health services research, from medical chart reviews to analysis of administrative datasets, to quantify the impact of avoidable hospital admissions, and indicate that a large proportion of hospital activity might in fact be avoidable. For the German health system context, Leutgeb *et al.* ^10^ demonstrated that hospital admission rates are much higher amongst nursing home residents compared to community-dwelling residents.

The number of people in need of nursing home care has steadily increased over 17% in the last ten years in Germany and, considering the country’s population is aging, it is expected to increase further. ^29^ Similar trends hold true for other high-income countries. ^30^

This is the first study to compare nursing home-sensitive with ambulatory care-sensitive conditions for the German setting. It showed that nursing home-sensitive conditions are to be distinguished from ambulatory care-sensitive conditions: 74% and 53% of common nursing home and nursing home-sensitive conditions, respectively, do not appear on the ambulatory care-sensitive list; for the core ambulatory care-sensitive list these numbers were 67% and 40%, respectively. In contrast, 69% of all ambulatory care-sensitive conditions did not appear on the list with 117 common nursing home conditions. Second, the number of hospitalizations that could have been prevented differed in case of optimal care conditions for the nursing home and outpatient setting, respectively. This may be due, in part, to the fact that Sundmacher *et al.* ^7^ estimated preventability for groups of ICD-10 conditions, clustered by disease category, while we estimated the preventability for every ICD-10 code individually. Although ICD-10 conditions and their estimated preventability differed in both health care settings, the main goal of these lists lies in raising awareness for which hospitalizations may be preventable.

The focus on nursing home-sensitive hospital admissions is therefore a critical issue for health care organization and reform. In addition to the financial implications associated with the potential of the diagnoses on our consensus list to avert hospitalization, reducing hospital admissions amongst nursing home residents would have a substantial impact on the person-centeredness of health care and quality of life of residents.

### Strengths and limitations

A strength of our study is that we were able to combine the analysis of routine health insurance data with a two-stage Delphi study and expert workshop. The health insurance data covered nearly 30% of the statutory health insured persons in Germany. For the Delphi study, we were able to recruit a high-calibre expert group and succeeded in ensuring a very high response rate of over 90%. Our subgroup analysis demonstrated that the assessment of experts was robust and not biased towards specialization, age or gender. This supports widespread recognition and applicability of our indicator list. By using the RAND/UCLA Appropriateness Method, ^22^ enhancing the Delphi method with an expert workshop, we were able to introduce direct interaction between experts as in other consensus development methods, ^31^ thereby combining the strengths of the Delphi methods with others.

The external validity of the results of the modified Delphi method, in general, is dependent on the representativeness of the panel of experts. The validity of the Delphi method depends, among other things, on the response rate, ^25^ with response rates between 51%-80% being recommended in the literature. ^32^^–^^35^ The commitment of participants to complete the Delphi process is often related to their interest and involvement with the question being examined. ^25^ In our study, we observed a high intrinsic motivation of the experts to participate, as many waived the incentive offered, and as evidenced by the extremely high response rate of 91%, with unlikely bias resulting from the minor loss-to follow-up. The validity of the Delphi method also depends on the included experts. ^26^ The more diverse and heterogeneous the expert panel is, the higher the quality of the decisions. ^36^ Four disciplines were represented in our panel, but no lay persons, such as nursing home residents or relatives. Another factor to consider when assessing the validity of the findings from the Delphi study is selection bias. If participants dropped out because of pseudonymity, there would only be a selection bias, if these individuals assessed the avoidance potential differently than the participants in this study. This is highly unlikely, as our results across Delphi rounds as well as various disciplines were very stable. The stability of the assessments in the individual Delphi rounds is considered more important than the response rate with regard to the occurrence of consensus. ^37^ We observed specialty-specific statistically significant, though minimal and therefore irrelevant, differences in estimated hospitalization preventability after the second round of surveys, for a subset (n= 34) of the 117 ICD-10 codes. Clinicians had slightly lower avoidability estimates, which could be due to the fact that they see the more serious cases in the clinic and are therefore more cautious with their assessment. Gender and age effects in estimating the proportion of potentially avoidable hospitalizations were low. Because of multidisciplinarity, the stable assessments of and the low dispersion in the estimates of the potential avoidability of hospitalization of nursing home residents and the relatively high number of included experts compared to other Delphi methods, it is rather unlikely that another panel would have come to different results. For these reasons, together with the high response rate, we expect the list of 58 nursing home-sensitive conditions to be generalizable.

The key limitation of our study, like comparable studies, is that preventability assessments were made assuming optimal structural and nursing home care conditions. We are aware that these conditions are currently not always met, and that interventions and improvement efforts are required to reduce or avoid hospital admission in the current health care setting.

### Researchers’ characteristics and contextual factors

All researchers were qualified health services researchers, familiar with the different methodological components of our study. However, the relationships between researchers and study participants were to a large extent limited to time-restricted conversations in a series of workshops. Given that participants were specialists invited for their specific expertise, researchers had no specific assumptions or presuppositions about the experts’ input. In the manuscript, no further interpretations of the experts’ inputs have been added.

The expert workshops and Delphi questionnaire – the only component of the research where qualitative comments were made – were all conducted online. No salient contextual factors impacting the research were identified.

### Implications for policy, research and clinical practice

Our study has various implications for policy, practice and research. Similar to the research on ambulatory care-sensitive hospital admissions, we assume that our study will lead to substantial debate and controversy about variations on nursing home-sensitive hospital admission rates and on the policy response to this variation. Such debate is likely to lead to proposals for improvements of the organization of the health care delivery system, and to constitute new indicators to monitor health system performance. For all stakeholders (e.g., medical and nursing providers, policy makers, health economists), the list of nursing home-sensitive conditions can inform the development of interventions and facilitate local quality improvement efforts. Practitioners and researchers should collaborate to identify the type of interventions (including personnel, staffing, skill mix, continuing professional education, infrastructure/resources, technology) required to reduce hospital admissions. Research should further address a costing of these interventions and calculations on the headroom (the maximum cost of the intervention to be cost-effective) to inform managers of nursing homes, hospitals and delivery systems. Finally, internationally comparative research should aim to identify a robust core basket of indicators for nursing home-sensitive hospital conditions for different health care system contexts. ^20^

## Data availability

All data that can be directly shared has already been included in the manuscript. Further, we have (i) used relevant keywords and descriptions for other researchers to identify our research (Findable), (ii) described our routes to data access for other researchers to grant access to similar data (Accessible), (iii) have used international nomenclature (ICD-10 codes) to facilitate merging of our datasets with those of other researchers (Interoperable) and (iv) we encourage other researchers to build on and reuse our methodological approach and data for further exploitation of the research findings (Reusable).

### Underlying data

Due to the provision of the data by the statutory health insurance (SHI) companies within the framework of a data evaluation contract, a direct publication is not possible. However, readers and reviewers may apply to access the data by contacting the following SHI companies. Several factors will be considered before access to data is granted, including the adherence to the EU General Data Protection Regulation.

- General Local Health Insurance Fund (AOK) in Rhineland/Hamburg ( aok@rh.aok.de)
- General Local Health Insurance Fund (AOK) Baden-Württemberg ( info@bw.aok.de)
- General Local Health Insurance Fund (AOK) Rhineland-Palatinate/Saarland ( service@rps.aok.de)
- BARMER Health Insurance Fund ( service@barmer.de)
- German Employees' Health Insurance Fund (DAK; service@dak.de)
- Health Insurance Fund (BKK) Werra-Meissner ( info@bkk-wm.de)

### Extended data

Open Science Framework: “Nursing home-sensitive conditions”, https://doi.org/10.17605/OSF.IO/EAJ58 ^38^

This project contains the following extended data:

- f1000Extended data_2021-11-03.pdf (questionnaire, workshop and data request documentation)

Data are available under the terms of the Creative Commons Attribution 4.0 International license (CC-BY 4.0).
