# Supplementary material for: Nursing home-sensitive conditions: analysis of routine health insurance data and modified Delphi analysis of potentially avoidable hospitalizations
Source: F1000Res. 2022 Apr 6;10:1223. Originally published 2021 Dec 1. [Version 2] doi: 10.12688/f1000research.73875.2 (PMC9021670; doi:10.12688/f1000research.73875.2)
Supplement: Peer review history

# Peer review: Reviewer response for version 2

**DOI:** 10.5256/f1000research.123399.r129944

**Published:** 2022-04-11

**Authors:** Daniela Holle

**Affiliations:** 1 Hochschule für Gesundheit, University of Applied Sciences, Bochum, Germany

Thank you for the opportunity to review the revised manuscript. The author group has commented comprehensively on all feedback and integrated the noted aspects into the article. I have no further comments.

 Best regards Daniela Holle

Is the work clearly and accurately presented and does it cite the current literature?

Partly

If applicable, is the statistical analysis and its interpretation appropriate?

Yes

Are all the source data underlying the results available to ensure full reproducibility?

Partly

Is the study design appropriate and is the work technically sound?

Partly

Are the conclusions drawn adequately supported by the results?

Partly

Are sufficient details of methods and analysis provided to allow replication by others?

Yes

Reviewer Expertise:

My focus is on nursing research, particularly in the area of nursing home care. I have no expertise in conducting economic studies.

I confirm that I have read this submission and believe that I have an appropriate level of expertise to confirm that it is of an acceptable scientific standard.

# Peer review: Reviewer response for version 1

**DOI:** 10.5256/f1000research.77561.r126098

**Published:** 2022-03-21

**Authors:** Tim Badgery-Parker

**Affiliations:** 1 Australian Institute of Health Innovation, Faculty of Medicine, Health and Human Sciences, Macquarie University, Sydney, NSW, Australia

This article describes the development of a list of "nursing home sensitive" conditions, and the use of the list to estimate costs of potentially preventable admissions from nursing homes to hospitals in Germany. The authors demonstrate differences between their list and an existing list of ambulatory sensitive conditions, confirming that the nursing home setting is sufficiently different, that the use of ambulatory care sensitive conditions is unlikely to be appropriate.

 The candidate diagnoses are derived from a large administrative dataset (30% of nursing home residents in Germany) and the process of developing the list is appropriate and comprehensively described, as is the cost estimation. The language is generally quite good, with some stray punctuation and a few unclear sentences.

 The results include a comparison of the preventability estimates between subgroups of the Delphi panel, this does not appear to be described in the methods.

 The results also describe criteria for excluding or combining diagnoses after the first round for presentation in the second round. (The criteria were not met for any diagnosis.)  These criteria should really be in the methods.

 Overall this is a strong study that makes a valuable contribution to the field.

Is the work clearly and accurately presented and does it cite the current literature?

Partly

If applicable, is the statistical analysis and its interpretation appropriate?

Yes

Are all the source data underlying the results available to ensure full reproducibility?

Yes

Is the study design appropriate and is the work technically sound?

Yes

Are the conclusions drawn adequately supported by the results?

Yes

Are sufficient details of methods and analysis provided to allow replication by others?

Yes

Reviewer Expertise:

I am a biostatistician with considerable experience in using administrative data to examine low-value hospital care and patient safety.

I confirm that I have read this submission and believe that I have an appropriate level of expertise to confirm that it is of an acceptable scientific standard.

# Response

**Published:** 2022-04-01

**Authors:** Sabine Bohnet-Joschko

**Affiliations:** Witten/Herdecke University, Germany

Dear editors, Dear Tim Badgery-Parker,

 We kindly thank you for your reply and for offering us the revision of our manuscript for consideration in the *F1000Research *Platform. We carefully considered the comments offered by you to improve the quality of the initial draft of the manuscript.

 Please find below a point-by-point response, in which we describe how we have addressed the suggestions. All changes or adjustments are shown in *italic font *and in revision mode in the revised paper accordingly.

 Again thank you for taking the time to critically review the paper. We truly appreciate the constructive input and hope that our revisions meet your approval.

 Yours sincerely, from the research team,

 Prof. Dr. Sabine Bohnet-Joschko

**Comment 1:**

 "The results include a comparison of the preventability estimates between subgroups of the Delphi panel, this does not appear to be described in the methods."

**Authors’ response to comment 1:**

 Thank you very much indeed for your review and the comments.

 We have now added two sentences to address this aspect:

 In the “Method” section, subsection “Delphi study and expert workshop”, fourth paragraph, last sentence:

*“…*and planned to recruit 100 experts: 30 outpatient/clinical physicians each, 30 nursing professionals and 10 scientists. *This also allowed for comparison of preventability assessments by expert group.*”

 And in the ninth paragraph:

 “In case the preventability potential of at least 75% was not comprised in the range of 75% of all expert assessments, the ICD-10 codes were excluded. For all other ICD-10 codes, statistical data (median, modal values, dispersion parameters, bar charts) *, results of expert group comparison on preventability assessments (Kruskal-Wallis (K-W)-Test), *and categorized comments from the Delphi questionnaire were provided for the expert workshop *.”*

**Comment 2:**

 "The results also describe criteria for excluding or combining diagnoses after the first round for presentation in the second round. (The criteria were not met for any diagnosis.) These criteria should really be in the methods."

**Authors’ response to comment 2:**

 Thank you for bringing this to our attention, which we, unfortunately, overlooked before. We have now moved the criteria to the methods section and only referred to them in the results section, as follows:

 In the “Method” section, subsection “Delphi study and expert workshop”, eighth paragraph, second sentence:

 “ *ICD-10 codes were planned to be excluded for assessment in the second round, when at least 75% of the experts estimated the preventability as zero in the first round. Secondly, ICD-10 codes were planned to be combined if the diseases were very similar (in terms of symptoms, diagnosis, prognosis, treatment) and the proportions of potential preventability were nearly identical. Two conditions for “nearly identical potential preventability” had to be fulfilled: the medians of the estimated preventability should not differ by more than 5% and the limits of the interquartile range had to be less than 10% apart. *Based on this information, *the ICD-10 codes for the second round were identified and *the participants were asked to quantify the proportion of potentially avoidable hospitalization again in the second round. *”*

 And in the “Results” section, subsection “Delphi study and expert workshop”, second paragraph, rather at the end, we changed the text "Furthermore, ICD-10 Codes... . Neither conditions were met sufficiently." was changed as follows:

*"The condition that at least 75% of the experts estimated the preventability as zero in the first round was met for none of the ICD-10 codes. Furthermore, none of the ICD-10 codes could be combined as the conditions for “nearly identical potential preventability” were not fulfilled."*

# Peer review: Reviewer response for version 1

**DOI:** 10.5256/f1000research.77561.r119120

**Published:** 2022-02-24

**Authors:** Daniela Holle

**Affiliations:** 1 Hochschule für Gesundheit, University of Applied Sciences, Bochum, Germany

Thank you for the option to review the manuscript entitled “Nursing home-sensitive conditions: analysis of routine health insurance data and modified Delphi analysis of potentially avoidable hospitalizations“.

 In a first research question, the study examines how often nursing home residents are treated in hospitals and what are the main diagnosis and associated costs of these hospital admissions. The second research question addresses potentially avoidable hospitals admission of nursing home residents. The third research question asks for the impact of the estimated preventability of hospital admissions. To answer the questions a secondary analysis of routine data based on health insurance claims data were used. In addition, a Delphi survey in combination with an expert workshop was conducted.

 In view of rising healthcare costs and increasingly scarce healthcare resources, answering the research questions is essential to ensure the best possible patient care while maintaining patient safety. A striking feature of the study is the very large data set of approximately €240,000 nursing home residents, which includes one-third of all statutorily insured nursing home residents. This is particularly noteworthy with regard to the representativeness of the study finding. The large number of experts who participated in the Delphi survey should also be emphasized.

 On closer inspection, however, I noticed a few aspects that I would like to briefly outline here:

 The introduction notes that interventions to reduce hospitalizations of nursing home residents should be tailored to health care systems. As an example of possible interventions, studies on the introduction and testing of nurse-led care models in nursing homes could be listed here. For example, the recent study by Zuniga *et al *(see Zúñiga *et al.* Positive effect of the INTERCARE nurse-led model on reducing nursing home transfers: A nonrandomized stepped-wedge design. ^1^) demonstrates that with an introduction of a nurse-led care model to hospital admissions of nursing home residents can be significantly reduced. Similar positive approaches can be found, for example, on the recent scoping review by Schmüdderich et al. (see Schmüdderich *et al.* Core elements and potential of nurse-led care models in residential long-term care: A scoping review. J Clin Nurs. 2022 Feb 4. ^2^). These efforts in the reduction of hospital admissions from nursing homes are worth noticing in the introduction.

 The methodology of the study is presented in a structured and comprehensible manner. However, from a content perspective, the study provides some limitations that I would like to outline briefly here and that should be critically presented and discussed in the course of the study.

 The routine data on discharge diagnoses were used to describe the admission diagnosis of nursing home residents. It should be critically reflected that there is a process between admission and discharge of a hospital patient, which can lead to confirmation, extension, or refutation of the admission diagnosis. Thus, the admission diagnosis is a limited indicator to describe the frequency and nature of hospital admissions of nursing home residents.

 With regard to the avoidability of hospital admissions, it must also be taken into account that the admission diagnosis alone is not necessarily the decisive factor for a corresponding admission, but also resident-specific criteria, which, in combination with a diagnosis, can lead to hospitalization. Thus, hospitalization may not be avoidable even with optimal care due to specific characteristics of nursing home residents.

 In this study, the significance of the results on the estimated avoidability of hospital admission mainly depends on the definition of optimal care for nursing home residents. In this manuscript, optimal care is defined by access to trained personnel, resources, and infrastructure for monitoring and nursing, and cooperation agreements with ambulatory general and specialists care providers (page 4/29, manuscript). Further explanations for the definition of optimal care are given within the attachment of the extended data (page 21/359):

- well-trained nursing staff
- the possibility or the equipment for a preliminary examination, for monitoring a possible crisis in the condition of the nursing home resident and for initiating treatment measures
- good cooperation with outpatient medical care
- the possibility of consultation with a specialist

In the definition of optimal care, it must be critically appreciated that both the qualification of the nursing staff and the cooperation of different professions were included, although well–trained outpatient medical care staff is also needed for optimal care.

 Nevertheless, the criteria for optimal care are very general, which is why a later discussion on the impact of avoidable hospital admissions also proves to be very difficult. With regard to the nursing staffing, the ratio between residents and nursing staff is relevant on the one hand, but also the skill and grade mix of the nursing staff against the background of the current resident structure in nursing homes. A good cooperation with the outpatient medical care could also have been specified in more detail, e.g. how often the individual resident is visited by an outpatient medical care service, how often the resident is also cared for by a specialist.

 The more specifically optimal care would have been described here, the more specifically the experts' assessments could have been estimated and the costs for the expenditure of optimal care could have been calculated and compared with the costs for preventable hospital admissions.

 In the course of the conclusion, it is stated that interventions should be developed that contribute to the reduction of hospitalizations of nursing home residents. In this context, it would be useful to refer to existing concepts (see comment introduction).

 I hope my comments help to strengthen the manuscript.

Is the work clearly and accurately presented and does it cite the current literature?

Partly

If applicable, is the statistical analysis and its interpretation appropriate?

Yes

Are all the source data underlying the results available to ensure full reproducibility?

Partly

Is the study design appropriate and is the work technically sound?

Partly

Are the conclusions drawn adequately supported by the results?

Partly

Are sufficient details of methods and analysis provided to allow replication by others?

Yes

Reviewer Expertise:

My focus is on nursing research, particularly in the area of nursing home care. I have no expertise in conducting economic studies.

I confirm that I have read this submission and believe that I have an appropriate level of expertise to confirm that it is of an acceptable scientific standard, however I have significant reservations, as outlined above.

## References

[1] : Positive effect of the INTERCARE nurse-led model on reducing nursing home transfers: A nonrandomized stepped-wedge design. *J Am Geriatr Soc* .2022; 10.1111/jgs.17677 10.1111/jgs.17677 35122238PMC9305956

[2] : Core elements and potential of nurse-led care models in residential long-term care: A scoping review. *J Clin Nurs* .2022; 10.1111/jocn.16231 10.1111/jocn.16231 35122351

# Response

**Published:** 2022-04-01

**Authors:** Sabine Bohnet-Joschko

**Affiliations:** Witten/Herdecke University, Germany

Dear Editors, Dear Daniela Holle,

 We kindly thank you for your reply and for offering us the revision of our manuscript for consideration in the *F1000Research *Platform. We carefully considered the comments offered by you to improve the quality of the initial draft of the manuscript.

 Please find below a point-by-point response, in which we describe how we have addressed the suggestions. All changes or adjustments are shown in *italic font *in the table below and in track changing mode in the revised paper accordingly.

 Again thank you for taking the time to critically review the paper. We truly appreciate the constructive input and hope that our revisions meet your approval.

 Yours sincerely, from the research team,

 Prof. Dr. Sabine Bohnet-Joschko

**Comment 1:**

 “As an example of possible interventions, studies on the introduction and testing of nurse-led care models in nursing homes could be listed here. For example, the recent study by Zuniga *et al *(see Zúñiga *et al.* Positive effect of the INTERCARE nurse-led model on reducing nursing home transfers: A nonrandomized stepped-wedge design.) demonstrates that with an introduction of a nurse-led care model to hospital admissions of nursing home residents can be significantly reduced. Similar positive approaches can be found, for example, on the recent scoping review by Schmüdderich et al. (see Schmüdderich *et al.* Core elements and potential of nurse-led care models in residential long-term care: A scoping review. J Clin Nurs. 2022 Feb 4.). These efforts in the reduction of hospital admissions from nursing homes are worth noticing in the introduction.”

**Authors’ response to comment 1:**

 Thank you for this valuable comment. We have now added the following sentence in the “Introduction” section, at the end of the second to last paragraph, and incorporated both references:

*“For instance, nurse-led care models with higher qualified nurses in expanded roles have been introduced and showed a positive impact on reducing hospitalization of nursing home residents and on advancing nursing practice in nursing homes, (Zúñiga et al. (2022), Schmüdderich et al. (2022)).”*

**Comment 2:**

 "The routine data on discharge diagnoses were used to describe the admission diagnosis of nursing home residents. It should be critically reflected that there is a process between admission and discharge of a hospital patient, which can lead to confirmation, extension, or refutation of the admission diagnosis. Thus, the admission diagnosis is a limited indicator to describe the frequency and nature of hospital admissions of nursing home residents."

**Authors’ response to comment 2:**

 Thank you for this comment: You are absolutely right, and therefore our study was based on discharge diagnoses of the nursing home residents instead of on admission diagnoses.

 To clarify this, we have added the following sentence to the “Methods” section, subsection “Analysis of routine health insurance data”, at the end of the second paragraph:

 “ *In line with previous studies, our analysis of routine health insurance data focused on the hospital discharge diagnosis, not on the admission diagnosis, as the latter is often subject to confirmation, extension or refutation during the hospital stay.*”

**Comment 3:**

 "With regard to the avoidability of hospital admissions, it must also be taken into account that the admission diagnosis alone is not necessarily the decisive factor for a corresponding admission, but also resident-specific criteria, which, in combination with a diagnosis, can lead to hospitalization. Thus, hospitalization may not be avoidable even with optimal care due to specific characteristics of nursing home residents."

**Authors’ response to comment 3:**

 This is absolutely correct and is well reflected by nursing home-sensitive conditions only expected to be avoidable in at least 70% of all cases with this condition. Conversely, this automatically means that up to 30% of the remaining cases with a disease represented in the nursing home-sensitive conditions catalogue are unavoidable hospitalizations.

 We included the following sentence under “Key findings” to clarify this:

 “After two Delphi rounds and an expert workshop, we were able to identify 58 diagnoses considered to be nursing home-sensitive conditions in the context of the German health care system *, i.e., at least seventy percent of all hospitalizations with such a condition is expected to be preventable, depending on the individual health status and advance directive of the nursing home resident*.”

**Comment 4:**

 In the definition of optimal care, it must be critically appreciated that both the qualification of the nursing staff and the cooperation of different professions were included, although well–trained outpatient medical care staff is also needed for optimal care.

 Nevertheless, the criteria for optimal care are very general, which is why a later discussion on the impact of avoidable hospital admissions also proves to be very difficult. With regard to the nursing staffing, the ratio between residents and nursing staff is relevant on the one hand, but also the skill and grade mix of the nursing staff against the background of the current resident structure in nursing homes. A good cooperation with the outpatient medical care could also have been specified in more detail, e.g. how often the individual resident is visited by an outpatient medical care service, how often the resident is also cared for by a specialist.

 The more specifically optimal care would have been described here, the more specifically the experts' assessments could have been estimated and the costs for the expenditure of optimal care could have been calculated and compared with the costs for preventable hospital admissions.

**Authors’ response to comment 4:**

 Thank you for this detailed evaluation of this aspect of our study. All mentioned aspects do matter indeed. We had some practical restraints, that made us decide to define optimal care conditions the way we did, which we have explained now in more detail in the “Discussion” section under “Strengths and limitations”:

*“The description of optimal care conditions was rather short, and perhaps therefore rather general. This could have led to less accurate preventability estimates. By addressing optimal care conditions in more detail (e.g., the importance of well-trained nursing staff both in the out- as well as the inpatient setting, special geriatric care knowledge and skills, resident structure in the nursing home, resident/ nursing staff ratio, skill and grade mix of staff, good cooperation with outpatient medical care, frequency of medical care visits by an outpatient medical care service/ specialist), experts might have given other (higher) estimates of preventability. This might have influenced the extrapolation of costs as well. As we already had a very long list of instructions, explanations and conditions to be assessed, we decided not to overstrain clarity and longevity for the Delphi experts hereupon, and kept the information provided on optimal care conditions to the point*.”

**Comment 5:**

 In the course of the conclusion, it is stated that interventions should be developed that contribute to the reduction of hospitalizations of nursing home residents. In this context, it would be useful to refer to existing concepts (see comment introduction).

**Authors’ response to comment 5:**

 We addressed this significant input in the “Discussion” section under “Implications for policy, research and clinical practice” as follows:

 “For all stakeholders (e.g., medical and nursing providers, policy makers, health economists), the list of nursing home-sensitive conditions can inform the development of interventions and facilitate local quality improvement efforts *, also taking into account existing evidence-based concepts.*”
